# Glycyrrhizin Ameliorates Radiation Enteritis in Mice Accompanied by the Regulation of the HMGB1/TLR4 Pathway

**DOI:** 10.1155/2020/8653783

**Published:** 2020-05-31

**Authors:** Xiao-min Zhang, Xiao Hu, Jin-ying Ou, Shan-shan Chen, Ling-hui Nie, Lei Gao, Ling-ling Zhu

**Affiliations:** ^1^School of Traditional Chinese Medicine, Southern Medical University, Guangzhou 510515, China; ^2^Traditional Chinese Pharmacological Laboratory, School of Traditional Chinese Medicine, Southern Medical University, Guangzhou 510515, China; ^3^Guangdong Traditional Medical and Sports Injury Rehabilitation Research Institute, Guangdong Second Provincial General Hospital, Guangzhou 510317, China; ^4^The Key Laboratory of Molecular Biology, State Administration of Traditional Chinese Medicine, School of Traditional Chinese Medicine, Southern Medical University, Guangzhou 510515, China; ^5^Department of Traditional Chinese Medicine, Nanfang Hospital, Southern Medical University, Guangzhou 510515, China

## Abstract

Radiation enteritis is a common side effect of radiotherapy for abdominal and pelvic malignancies, which can lead to a decrease in patients' tolerance to radiotherapy and the quality of life. It has been demonstrated that glycyrrhizin (GL) possesses significant anti-inflammatory activity. However, little is known about its anti-inflammatory effect in radiation enteritis. In the present study, we aimed to investigate the potential anti-inflammatory effects of GL on radiation enteritis and elucidate the possible underlying molecular mechanisms involved. The C57BL/6 mice were subjected to 6.5 Gy abdominal X-ray irradiation to establish a model of radiation enteritis. Hematoxylin and eosin staining was performed to analyze the pathological changes in the jejunum. The expression of TNF-*α* in the jejunum was analyzed by immunochemistry. The levels of inflammatory cytokines, such as TNF-*α*, IL-6, IL-1*β*, and HMGB1 in the serum were determined by enzyme-linked immunosorbent assay. The intestinal absorption capacity was tested using the D-xylose absorption assay. The levels of HMGB1 and TLR4 were analyzed by western blotting and immunofluorescence staining. We found that GL significantly alleviated the intestinal damage and reduced the levels of inflammatory cytokines, such as TNF-*α*, IL-6, IL-1*β*, and HMGB1 levels. Furthermore, the HMGB1/TLR4 signaling pathway was significantly downregulated by GL treatment. In conclusion, these findings indicate that GL has a protective effect against radiation enteritis through the inhibition of the intestinal damage and the inflammatory responses, as well as the HMGB1/TLR4 signaling pathway. Thereby, GL might be a potential therapeutic agent for the treatment of radiation enteritis.

## 1. Introduction

Radiotherapy is one of the effective methods for the treatment of malignant abdominal and pelvic tumors [1, 2]. However, it serves as a double-edged sword. Although abdominal irradiation is aimed to target the malignant tissues, the adjacent healthy organs can be greatly influenced as well, especially the rapidly renewing organs involving the epithelial cells of the small intestine [3, 4]. The small intestine is considered to be one of the most sensitive tissues in the abdomen to radiation exposure. After transient radiation exposure, ROS and NOS are immediately generated [5–7], which can cause severe cell damage, including DNA damage and the release of intracellular cytokines [8, 9]. Radiation-induced DNA damage can act as a damage-associated molecular pattern (DAMP) to stimulate inflammatory responses, as well as destruction of the intestinal epithelial barrier mediated by abdominal radiation exposure, ultimately resulting in radiation enteritis [5, 9–11].

High-mobility group box 1 (HMGB1) protein is an important member of the DAMP family, which is translocated from the nucleus to the cytoplasm and released into the extracellular environment in response to cell stress, damage, and death, thereby acting as an important endogenous danger signal and a crucial proinflammatory mediator [12, 13]. When HMGB1 binds to the toll-like receptor 4 (TLR4) or the receptor for advanced glycation end products (RAGE), it triggers inflammatory responses and then activates the release of cytokines, leading to further tissue injuries [14]. As an early mediator of inflammation, HMGB1 is implicated in a variety of inflammatory diseases, including sepsis [15], arthritis and pneumonia [16, 17], thereby indicating that HMGB1 maybe be a promising therapeutic target for inflammatory diseases [18].

TLR4 belongs to the family of toll-like receptors (TLRs), which recognize exogenous pathogen-associated molecular patterns (PAMPs) and endogenous DAMPs [19]. TLR4 exists in a variety of tissues, including the brain [20], myocardium, and small intestine [21]. Once TLR4 recognizes HMGB1 released from the damaged tissues, the production of cytokines, such as IL-6, TNF-*α*, and IL-1*β* is activated to amplify the inflammatory cascade [22, 23]. Recently, it has been reported that the HMGB1/TLR4 pathway aggravates the destruction of intestinal mucosa in colitis [24]. Nevertheless, the role of HMGB1/TLR4 pathway in radiation enteritis has not been explored.

Glycyrrhizin (GL), the main active constituent derived from *Glycyrrhizaglabra*, exerts a variety of pharmacological effects, including anti-inflammatory [25, 26], anti-oxidant, anti-tumor, and hepatoprotective effect [27]. It has been proved that GL is an effective inhibitor of HMGB1 by many studies [28, 29]. Clinically, GL has been commonly used in the treatment of chronic hepatitis in Japan [30]. However, there is no study on its effect on radiation enteritis. Therefore, in this study, we investigated whether GL could ameliorate radiation enteritis by regulating the HMGB1/TLR4 pathway.

## 2. Materials and Methods

### 2.1. Animals

A total of 40 specific-pathogen-free male C57BL/6 mice (weighing 18–22 g and aged 8 weeks) were obtained from the Southern Medical University Experimental Animal Center (certificate number was SYXK Guangdong 2016-0167). The mice were acclimatized to standard laboratory conditions with 24 ± 2°C, 55–60% humidity and a 12-hour light/dark cycle. All animal experiments were performed strictly in accordance with the Guide for the Care and Use of Laboratory Animals by the National Institutes of Health and were approved by the Southern Medical University's Experimental Animal Ethics Committee.

### 2.2. Glycyrrhizin Treatment

Glycyrrhizin (purity ≥98.0%) was purchased from MedChemExpress (Shanghai, China). All compounds were dissolved in normal saline containing 10% DMSO. A total of 40 mice were randomly divided into five groups, namely the control, model, low-dose GL (5 mg/kg/day, i.p.), medium-dose GL (10 mg/kg/day, i.p.), and high-dose GL (20 mg/kg/day, i.p.) groups, with eight mice in each group. The mice were exposed to a single dose of 6.5 Gy abdominal X-ray irradiation using an irradiator instrument MultiRad (MultiRad 225, Faxitron, USA) at a dose rate of 0.99 Gy/min. To limit the exposure of the bone marrow to irradiation, the head, chest, and extremities of mice were shielded with lead strips. The mice in the control group were sham-irradiated. Two hours before irradiation, the mice in the treatment group were pretreated with different doses of GL (5 mg/kg, 10 mg/kg, and 20 mg/kg). Within two hours after irradiation, the mice were intraperitoneally (i.p.) administrated with the previous doses of GL for three consecutive days. The body weight of mice in each group was monitored every day.

### 2.3. Histopathological Examination and Immunochemistry

For the histopathological examination, all mice were sacrificed 3.5 days after irradiation. The jejunum was collected and fixed in 4% paraformaldehyde for 48 hours and then embedded in paraffin. Sections were cut in 4 *μ*m of thickness and used for hematoxylin and eosin (H&E) staining. For immunochemical analysis, paraffin sections were boiled in sodium citrate to repair antigen. Then, the endogenous peroxidase enzyme was inactivated using a 3% H_2_O_2_ solution for 10 min. After three washes with PBS, the sections were sealed with 5% nonfat dry milk in TBST for 2 h and then incubated with the primary antibody diluted in 5% nonfat dry milk overnight at 4°C. The primary antibody used in immunochemistry was antibody against TNF-*α* (Rabbit, 1 : 250, Abcam). After three washes with PBS, the sections were incubated with a biotinylated goat anti-rabbit secondary antibody for 2 h at room temperature. Afterward, the sections were stained with DAB for 5–10 min and counterstained with hematoxylin. These sections were visualized using an optical microscope (Olympus IX53; Olympus, Japan).

### 2.4. Serum Analysis Using Enzyme-Linked Immunosorbent Assay (ELISA)

Serum concentrations of TNF-*α*, IL-6, IL-1*β*, and HMGB1 were determined by using the commercial ELISA kits (R&D Systems) and the protocols were adopted according to the manufacturer's instructions.

### 2.5. D-Xylose Absorption Assay

The mice were orally administered with a 5% w/v solution of D-xylose (100 *μ*L/mouse) in deionized water. After 2 hours of D-xylose administration, the mice were sacrificed and blood samples were collected by heparinized blood tubes. Then 50 *μ*L of serum sample was added to 5 mL of phloroglucinol colour reagent, and heated to 100°C in a water bath for 4 min. After equilibration to room temperature, D-xylose absorption was measured with the aid of a spectrophotometer set at 554 nm.

### 2.6. Western Blotting Analysis

The jejunum of each group was lysed in RIPA lysis buffer (Thermo Fisher Scientific, USA) containing protease inhibitor and phosphatase inhibitor. The total protein concentration was determined using the BCA protein estimation kit (Thermo Fisher Scientific, USA). Equal quantities of the total protein were loaded into wells and separated using SDS-PAGE, and then transferred to polyvinylidene fluoride membranes. The membranes were blocked with 5% nonfat dry milk in TBST at room temperature for 1 h, and incubated with primary antibodies overnight at 4°C. After three washes in TBST, the membranes were incubated with goat anti-rabbit secondary antibody conjugated with horseradish peroxidase (1 : 2000) at 4°C for 2 h. Immune-reactive proteins were detected by ECL reagents (Thermo Fisher Scientific, USA). The following primary antibodies were used in western blotting: Antibodies against HMGB1 (Rabbit, 1 : 1000, Abcam), TLR4 (Rabbit, 1 : 500, Abcam), and GAPDH (Rabbit, 1 : 1000, Cell Signaling Technology). GAPDH functioned as an internal reference and then quantification analysis was performed using the ImageJ (NIH) software.

### 2.7. Immunofluorescence Analysis

After the mice were sacrificed, the jejunum was collected and fixed in 4% paraformaldehyde for 48 h, and then in 30% sucrose solution for 72 h. The jejunum was cut into 8 *μ*m sections and infiltrated with 0.1% Triton X-100 in PBS for 30 min, and then incubated with the blocking solution containing 5% nonfat dry milk at room temperature for 2 h. Primary antibodies were added to the sections and incubated overnight at 4°C. The following primary antibodies were used: HMGB1 (Rabbit, 1 : 250, Abcam) and TLR4 (Rabbit, 1 : 100, Abcam). After washing with PBS, the sections were incubated with anti-rabbit secondary antibodies at room temperature for 1 h. The following secondary antibodies were used, goat anti-rabbit Alexa Fluor 488-conjugated IgG (1 : 500, Life Technologies) and goat anti-rabbit Alexa Fluor 594-conjugated IgG (1 : 500, Life Technologies). Then, the sections were counterstained with DAPI for 5 minutes. After washing twice with PBS, the sections were covered with the fluorescent mounting medium (Solarbio) and coverslip. Finally, the sections were visualized under a fluorescence microscope (Olympus, Tokyo, Japan).

### 2.8. Statistical Analysis

Statistical analysis was performed using the GraphPad Prism software (ver. 5.0 GraphPad Software, San Diego, USA). The data were analyzed using one-way ANOVA followed by Tukey's multiple comparison tests and shown as mean ± SEM. Data were representative of three independent experiments. *P* < 0.05 was considered as a statistically significant difference.

## 3. Results

### 3.1. Glycyrrhizin Increases the Body Weight of C57BL/6 Mice with Radiation Enteritis

**Figure 1:**
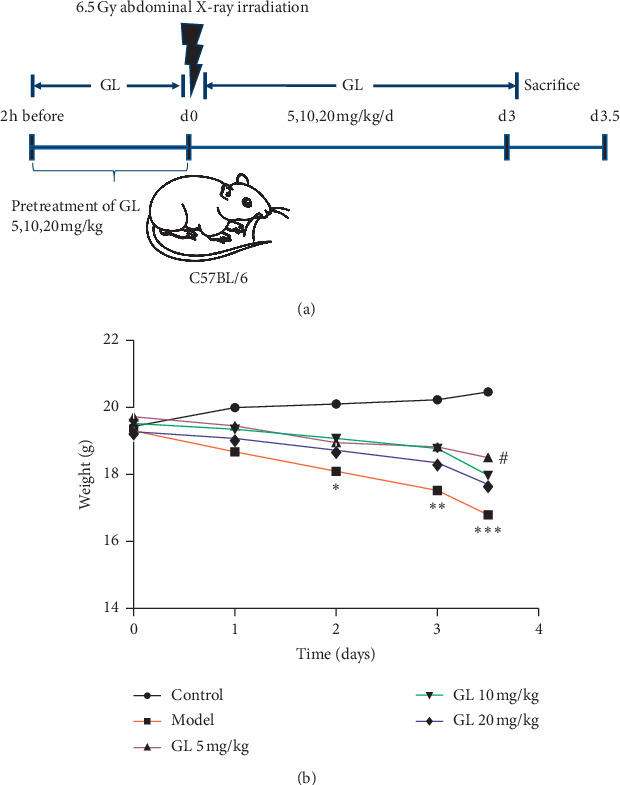
Glycyrrhizin increased the body weight of C57BL/6 mice with radiation enteritis. (a) Establishment of a model of radiation enteritis with a total dose of 6.5 Gy x-rays and the GL treatment protocols for C57BL/6 mice with radiation enteritis, which were administered with GL (5 mg/kg, 10 mg/kg, and 20 mg/kg) for three days. (b) Body weight of each group was monitored per day. ^*∗*^*P* < 0.05, ^*∗∗*^*P* < 0.01, and ^*∗∗∗*^*P* < 0.001 vs. the control group; ^#^*P* < 0.05 vs. the model group.

To investigate the potential anti-inflammatory effects of GL on radiation enteritis, we established a mouse model of radiation enteritis with a total dose of 6.5 Gy X-ray irradiation. Two hours before or after irradiation, the mice were treated with different doses of GL (5 mg/kg, 10 mg/kg, 20 mg/kg) for three days (Figure 1(a)).  Figure 1(b) shows changes in the body weight within 3.5 days for all groups. The mice with radiation enteritis showed a significant decrease in body weight on day 2 (Figure 1(b). In comparison with the model group, the mice treated with GL (5 mg/kg) showed reduced weight loss on day 3.5 (Figure 1(b).

### 3.2. Glycyrrhizin Alleviates the Jejunum Pathology

**Figure 2:**
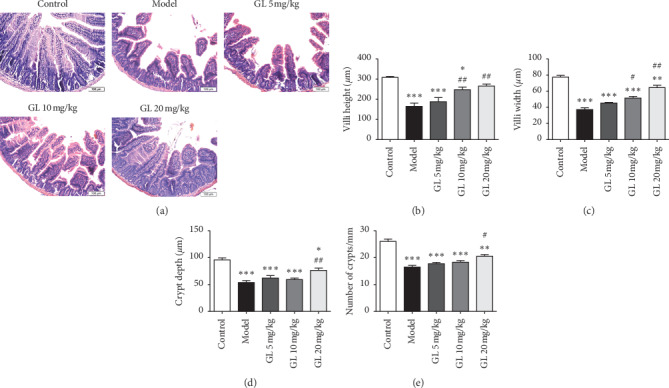
GL significantly improved the jejunum pathology in C57BL/6 mice with radiation enteritis. (a) Representative jejunum histopathology (H&E staining) of each group. All magnifications: ×200. (b–e) Intestinal villus height, villus width, crypt depth, and crypt count of each group were measured to evaluate the effect of GL on intestinal morphology. ^*∗*^*P* < 0.05, ^*∗∗*^*P* < 0.01, and ^*∗∗∗*^*P* < 0.001vs. the control group; ^#^*P* < 0.05 and ^##^*P* < 0.01 vs. the model group.

Pathological examination of H&E-stained jejunum showed that the morphology of jejunum rapidly changed after irradiation, manifested by loss of intestinal epithelial integrity, villi denudation, and mucosal muscular layer thinning. Interestingly, the mice administered with 20 mg/kg GL exhibited relatively well-preserved histological architecture with less intestinal epithelium damage (Figure 2(a)). H&E staining of the jejunum showed that the mice exposed to abdominal irradiation exhibited evidently decreased villus height, villus width, crypt depth, and crypt count compared to the control mice (Figures 2(b)–2(d). Increased villus height, villus width, crypt depth, and crypt count were observed in C57BL/6 mice administered with 20 mg/kg GL compared to that in the model mice (Figures 2(b)–2(d).

### 3.3. Glycyrrhizin Downregulates Proinflammatory Cytokines Levels

**Figure 3:**
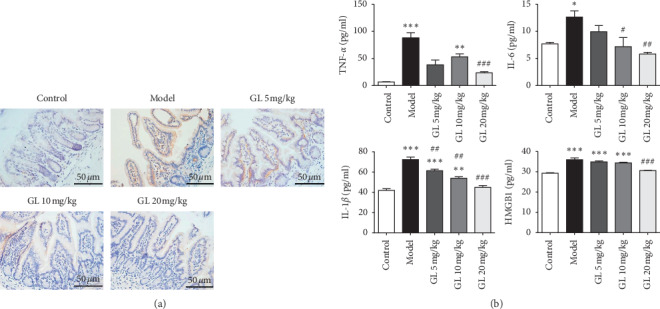
GL exerted anti-inflammatory activity by downregulating the levels of proinflammatory cytokines TNF-*α*, IL-6, IL-1*β*, and HMGB1. ((a) The expression of TNF-*α* in the jejunum was detected by immunochemistry. ((b) The levels of proinflammatory cytokine TNF-*α*, IL-6, IL-1*β*, and HMGB1 in serum were measured by ELISA to assess different doses of GL anti-inflammatory activity. ^*∗*^*P* < 0.05, ^*∗∗*^*P* < 0.01, and ^*∗∗∗*^*P* < 0.001 vs. the control group; ^#^*P* < 0.05, ^##^*P* < 0.01, and ^###^*P* < 0.001 vs. the model group.

To explore the anti-inflammatory effects of GL, the expression of TNF-*α* in the jejunum was measured by immunochemistry, and the levels of inflammatory cytokines, such as TNF-*α*, IL-6, IL-1*β*, and HMGB1 in serum, were determined by ELISA kits. Immunochemistry results revealed that GL could inhibit the expression of TNF-*α* in the jejunum (Figure 3(a)). Moreover, the serum levels of cytokines, TNF-*α*, IL-6, IL-1*β*, and HMGB1 were significantly increased in the model mice (Figure 3(b). However, 20 mg/kg GL treatment markedly reduced the levels of inflammatory cytokines, TNF-*α* and HMGB1 in the mice with radiation enteritis (Figure 3(b). In addition, GL inhibited the levels of IL-6 and IL-1*β* in a dose-dependent manner (Figure 3(b). These results indicate that GL plays a significant anti-inflammatory role in radiation enteritis.

### 3.4. Glycyrrhizin Ameliorates Intestinal Absorption

**Figure 4:**
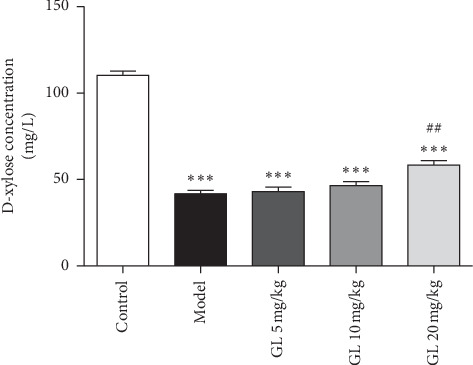
Glycyrrhizin ameliorated the intestinal absorption capacity. D-xylose absorption test was used to assess the effect of GL on intestinal absorption. There was a significantly decreased level of D-xylose in the model group. In contrast, there was an increased level of D-xylose in the mice administered with 20 mg/kg GL. ^*∗∗∗*^*P* < 0.001 vs. the control group; ^##^*P* < 0.01 vs. the model group.

To evaluate the absorptive capacity of the intestine after radiation exposure, the mice were fed with a D-xylose solution. Because D-xylose is not metabolized in the body, serum D-xylose level can well reflect the intestinal absorption capacity. There was a significant reduction in the D-xylose level in the model group 3.5 days after irradiation exposure. On the contrary, there was an increased level of D-xylose in the mice administered with 20 mg/kg GL (Figure 4).

### 3.5. Glycyrrhizin Inhibits the HMGB1/TLR4 Pathway in Mice with Radiation Enteritis

**Figure 5:**
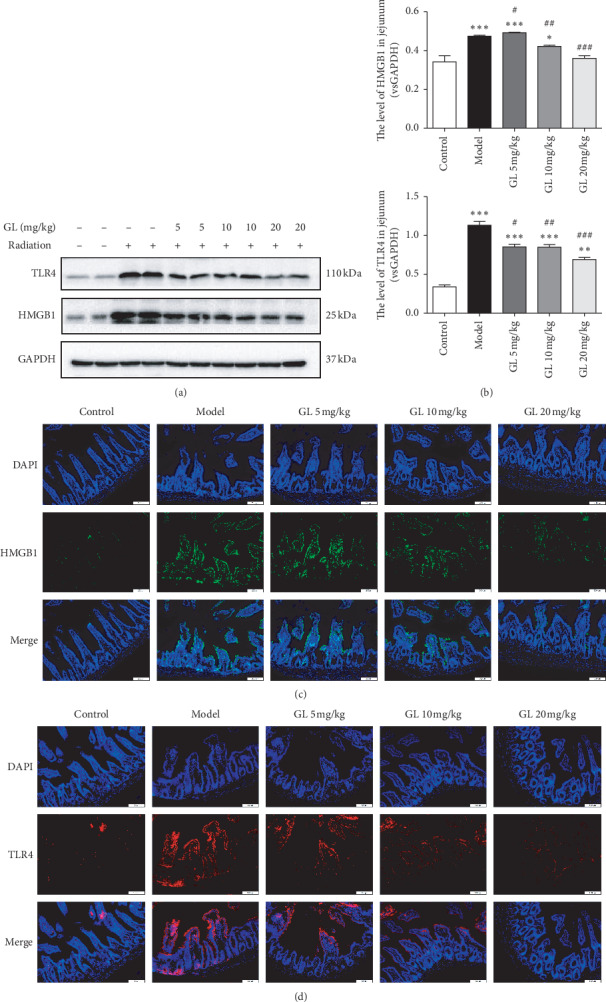
GL inhibited the expression of HMGB1 and TLR4 in the jejunum by western blotting analysis and immunofluorescence analysis. (a) Representative western blotting bands of HMGB1 and TLR4 proteins in the jejunum. (b) The target protein/GAPDH ratios, including HMGB1/GAPDH and TLR4/GAPDH ratios. (c and d) The expression and localization of HMGB1 and TLR4 in the jejunum were analyzed by immunofluorescence. All magnifications: ×200.^*∗*^*P* < 0.05, ^*∗∗*^*P* < 0.01, and ^*∗∗∗*^*P* < 0.001 vs. the control group; ^#^*P* < 0.05, ^##^*P* < 0.01, and^###^*P* < 0.001 and vs. the model group.

Since GL exhibited markedly anti-inflammatory effects in radiation enteritis (Figure 3), we further evaluated the effects of GL on HMGB1/TLR4 pathway by western blotting analysis and immunofluorescence analysis, which is known to play a crucial role in various inflammatory diseases. The elevation of HMGB1 and TLR4 expression was detected in the model group (Figure 5). Interestingly, the result of the western blotting analysis revealed that GL treatment significantly decreased the expression of HMGB1 and TLR4 in C57BL/6 mice with radiation enteritis, and administered with 20 mg/kg GL reduced HMGB1 and TLR4 expression most effectively (Figure 5). These results were consistent with the findings of intestinal immunofluorescence staining. Immunofluorescence staining showed that GL downregulated the expression of HMGB1 and TLR4 in radiation enteritis (Figures 5(c) and 5(d)). Furthermore, the results revealed that the secretion of HMGB1 was at the tips of the villi (Figure 5(c)).

## 4. Discussion

Radiotherapy is one of the effective treatments for abdominal and pelvic malignancies. However, radiation enteritis not only reduces patients' quality of life but also reduces the tolerance to radiotherapy [31, 32]. Due to the lack of drugs particularly effective in the prevention and treatment of radiation enteritis, new agents are urgently needed. It has been reported that GL could alleviate inflammatory responses and was adopted as a therapeutic agent in a variety of inflammatory diseases, for instance cerebral ischemia/reperfusion-induced inflammation and arthritis [33]. Therefore, in our present study, we evaluated the potential anti-inflammatory effects of GL on radiation enteritis.

The mice were subjected to 6.5 Gy abdominal X-ray irradiation to establish a model of radiation enteritis. When the small intestine is exposed to irradiation, inflammatory responses are activated immediately followed by the generation of cytokines. In recent studies, intestinal epithelial cells were identified as the main source of HMGB1, which participated in a variety of inflammatory responses [34, 35]. It is well known that TNF-*α* and IL-6 are produced in a variety of inflammatory responses. In this study, the administration of GL markedly decreased the expression of TNF-*α* in the jejunum and the levels of TNF-*α*, IL-6, IL-1*β*, and HMGB1 in serum. This indicates that GL exerts the anti-inflammatory activity in radiation enteritis, which was consistent with the findings of the studies on murine colitis and sepsis [36, 37]. In addition, the results showed that the GL treatment improved the villus height, the villus width, the crypt depth, and the crypt count, as well as the level of D-xylose in serum after radiation exposure, which indicates that GL has a protective effect on intestinal damage caused by irradiation. However, because the mice were subjected to the sublethal dose of radiation in the current study, they rarely died of severe gastrointestinal syndrome within 3.5 days. Thus, the study lacks the exploration of the effect of GL on the survival of mice, indicating a higher dose of radiation is needed to investigate the effect of GL on the survival of mice.

As a major member of DAMPs, HMGB1 plays a crucial role in the immune or inflammatory responses. Once HMGB1 binds to TLR4, the inflammatory response is activated that subsequently induces the expression of cytokines. Thus, inhibition of the HMGB1/TLR4 pathway shows potential anti-inflammatory effects [18]. Furthermore, several previous studies have demonstrated that GL significantly downregulates the HMGB1/TLR4 pathway [38–40]. Therefore, the expression levels of HMGB1 and TLR4 were evaluated in our experiments. In our study, the HMGB1/TLR4 pathway was upregulated in the model group and the administration of GL significantly inhibited the HMGB1/TLR4 pathway. These results indicate that GL could protect the jejunum against radiation enteritis by regulating the HMGB1/TLR4 pathway. In addition, intestinal epithelial cells can produce citrulline, which has been considered as a negative regulator of TLR4 signal and a sensitive biomarker for radiation-induced intestinal injury [41, 42]. Thereby, these suggest that the protective effect of GL against radiation enteritis may be related to the regulation of citrulline. Meanwhile, we found that the secretion of HMGB1 in radiation enteritis was polarized, and released to the tips of the villi rather than the basolateral environment. It is possible due to the fact that TLR4, which functions as a key receptor for HMGB1, is located on the apical surface of the intestinal epithelial cells [43].

Furthermore, it must be noted that this preliminary study presented here, lacks an exploration of other important factors that mediate radiation enteritis, such as the destruction of the intestinal epithelial barrier and tight junction after irradiation exposure [12, 44]. Besides, irradiation exposure not only acts as a proinflammatory signal [5, 45], but also mediates oxidative and nitrative stress to induce the production of ROS and NOS in the small intestine [7, 9, 46, 47]. In addition, GL has been reported to possess anti-oxidant properties in a recent study [48]. Thus, aside from its anti-inflammatory effects, GL might protect the jejunum against radiation-induced injury by ameliorating oxidative stress, which remains to be further explored in the future. It is reported that the high level of HMGB1 promotes the migration, invasion, and autophagy of cancer cells, which was inhibited by GL [49, 50], indicating that GL may exert an antitumor effect and possibly improve the efficacy of radiotherapy in the treatment of the tumors.

## 5. Conclusion

In conclusion, the findings of our study indicate that GL exerts anti-inflammatory activity by downregulating the levels of proinflammatory cytokines and alleviates intestinal damage in mice with radiation enteritis, accompanied by the downregulation of the HMGB1/TLR4 pathway. Therefore, GL might be a promising therapeutic agent for the treatment of radiation enteritis. Although the current study has shown the initial changes in radiation enteritis and HMGB1/TLR4 signaling after GL treatment, further studies are still needed to elucidate the underlying mechanisms involved. Moreover, the study indicates that GL has a protective effect on intestinal damage after radiation, but the influence of GL treatment on the efficacy of radiotherapy in the treatment of the tumor needs to be evaluated.

## Data Availability

The data used to support the findings of this study are included within the article.
